# Changes in Symptoms and Lung Function During Three Months of Individually Tailored Inhaled Therapy in Chronic Obstructive Pulmonary Disease: A Prospective Observational Cohort Study

**DOI:** 10.3390/jcm15197722

**Published:** 2026-10-07

**Authors:** Bektas Isik, Hilmi Erdem Sumbul, Huseyin Ali Ozturk, Begum Seyda Avci, Erdinc Gulumsek, Merve Sumbul, Irfan Alisan, Ahmet Gazi Mustan, Fatih Necip Arici, Cisem Yilmaz, Cahit Dincer, Okan Pirinci, Ramazan Azim Okyay

**Affiliations:** 1 Department of Internal Medicine, University of Health Sciences, Adana Health Practice and Research Center, Adana 01230, Türkiye; 2 Department of Family Medicine, Adana City Research and Training Hospital, Adana 01230, Türkiye; 3 Department of Public Health, Adana City Research and Training Hospital, Adana 01230, Türkiye

**Keywords:** administration, inhalation, bronchodilator agents, pulmonary disease, chronic obstructive, patient reported outcome measures, treatment outcome

## Abstract

**Background.** Randomised controlled trials have established what individual inhaled regimens can achieve in chronic obstructive pulmonary disease (COPD) under controlled conditions, but they recruit selected populations and enforce protocol-driven follow-up. How patients fare on regimens chosen individually for them in everyday care is less well characterised. **Objective.** To assess the change in symptoms and lung function over three months in a prospective cohort of patients with COPD whose inhaled treatment was tailored to the individual by the attending physician. **Methods.** Consecutive adults with stable, spirometrically confirmed COPD attending a single internal-medicine clinic were assessed at baseline and reassessed approximately three months later. Baseline regimens were short-acting beta_2_-agonist/short-acting muscarinic antagonist (SABA/SAMA), long-acting muscarinic antagonist (LAMA), long-acting beta_2_-agonist (LABA), LABA plus inhaled corticosteroid (ICS), LABA plus LAMA, or LABA plus LAMA plus ICS. The primary outcomes were the within-participant change in COPD Assessment Test (CAT) score and in forced expiratory volume in one second (FEV_1_) % predicted. Secondary outcomes were the proportion achieving the CAT minimal clinically important difference (MCID, defined as a reduction of at least two points), the proportion achieving a six-minute walk distance (6MWD) improvement of at least 30 m, change in modified Medical Research Council (mMRC) dyspnoea grade, body mass index–airflow obstruction–dyspnoea–exercise capacity (BODE) and age–dyspnoea–obstruction (ADO) indices, and clinical events. Paired changes were analysed with paired *t*-tests or Wilcoxon signed-rank tests and are reported with 95% confidence intervals (CIs). **Results.** Three hundred participants were enrolled. Recorded GOLD groups were A (*n* = 69), B (n = 158) and E (n = 73). Paired CAT and FEV_1_ data were available for 276 (92.0%) and 257 (85.7%) participants, all of whom were reassessed between 84 and 98 days after baseline (median 91 days). Across the cohort, mean CAT fell by 3.93 points (95% CI −4.43 to −3.44) and mean FEV_1_ rose by 2.20 percentage points (95% CI 1.78 to 2.63); one hundred and ninety-five of 276 participants (70.7%) reached the CAT MCID and 98 of 244 (40.2%) achieved a 6MWD gain of at least 30 m. Improvement was observed with every long-acting regimen. Participants receiving LABA + ICS improved by 4.18 CAT points (95% CI −5.24 to −3.12; *p* < 0.001) and 3.06 FEV_1_ percentage points (95% CI 2.16 to 3.95; *p* < 0.001), with 82.1% reaching the CAT MCID—a magnitude similar to that observed on LABA+LAMA. Participants on SABA/SAMA alone showed no improvement in either outcome. Sixty-three exacerbations, eleven hospitalisations and four cases of pneumonia occurred. **Conclusions.** During a reassessment interval of 84 to 98 days, patients with COPD receiving individually tailored long-acting inhaled therapy showed improvement in symptoms and lung function that exceeded the established MCID for the CAT, including patients treated with LABA+ICS. The study had no concurrent control group, no randomisation and no adjustment for baseline differences between regimens; the observed changes therefore describe the course of treated patients and cannot be attributed to any specific regimen.

## 1. Introduction

Chronic obstructive pulmonary disease (COPD) remains a leading cause of death and disability worldwide, and its burden continues to shift across regions and age structures [1]. Management is now guided by a multidimensional assessment that combines spirometry with symptom scores, exacerbation history, blood eosinophil count and comorbidity [2,3], and patients are grouped as A, B or E according to symptom burden and exacerbation risk [4].

The strongest evidence on inhaled treatment in COPD comes from large randomised controlled trials. Dual bronchodilation with a long-acting beta_2_-agonist (LABA) plus a long-acting muscarinic antagonist (LAMA) reduced exacerbations more than a LABA plus inhaled corticosteroid (ICS) combination in FLAME [5], and triple therapy outperformed dual therapy in IMPACT and ETHOS [6,7]. On this basis, current guidance positions dual long-acting bronchodilation as the usual starting point and reserves ICS-containing regimens for selected patients [2,8]. These trials, however, enrol populations defined by strict eligibility criteria, standardise the device and the molecule, and enforce protocol-driven adherence monitoring. They therefore describe efficacy under controlled conditions rather than the change a physician can expect in an unselected clinic population whose treatment is chosen individually, one patient at a time.

Symptom impact is most often quantified with the COPD Assessment Test (CAT) [9], which the 2026 GOLD report refers to as the Chronic Airways Assessment Test, a renaming that leaves the items and scoring unchanged [10]. A reduction of approximately two points is the accepted minimal clinically important difference (MCID) [11]. Multidimensional indices such as the body mass index–airflow obstruction–dyspnoea–exercise capacity (BODE) index [12] and the age–dyspnoea–obstruction (ADO) index [13] add prognostic information.

Everyday practice differs from trial conditions in case mix, adherence, device familiarity and concurrent care. Observational studies addressing this gap remain relatively few and are dominated by single-inhaler triple therapy [14,15]. Data from internal-medicine settings, where a substantial share of COPD care is delivered in many health systems, are particularly scarce.

### Objective

The objective of this study was to assess the change in symptoms, lung function, exercise capacity and multidimensional indices over an approximately three-month interval in a prospective cohort of patients with COPD whose inhaled treatment was tailored to the individual by the attending physician, overall and within each GOLD group and each regimen. This is a longitudinal observational study of within-patient change; it was not designed, and is not presented, as a comparative assessment of the efficacy of one regimen against another.

## 2. Materials and Methods

### 2.1. Study Design and Setting

This prospective, single-centre, observational cohort study was conducted in the Department of Internal Medicine, University of Health Sciences—Adana Health Practice and Research Center, Adana, Türkiye. Consecutive adults attending the stable-COPD clinic between 1 March 2026 and 1 September 2026 were screened. Reporting follows the STROBE recommendations [16].

Sampling was consecutive and non-probabilistic: every eligible patient attending the clinic during the recruitment window was invited, and recruitment stopped when the window closed. The cohort is therefore representative of the COPD population attending this internal-medicine clinic, and not of the general COPD population of Türkiye. No formal power calculation was performed, because the study estimates within-patient change rather than testing a between-group hypothesis; the target of 300 participants was set by the number of patients the clinic could assess and reassess with full spirometry and six-minute walk testing during the recruitment period. No randomisation, stratification or matching was used at any stage, and no between-arm event rates were anticipated, since the study has no arms in the trial sense.

### 2.2. Participants

Eligible participants were aged ≥40 years, had stable COPD with no exacerbation in the preceding four weeks, a post-bronchodilator FEV_1_/forced vital capacity (FVC) ratio below 0.70, and provided written informed consent. Participants with a physician diagnosis of asthma or asthma–COPD overlap, an acute exacerbation at enrolment, or active malignancy were excluded. Active malignancy was identified from the hospital electronic medical record and the patient interview at enrolment; no additional screening investigations were performed for study purposes. No participant was excluded on the basis of diabetes mellitus, chronic kidney disease, adrenal insufficiency or other stable comorbidity, because the study was designed to reflect the comorbidity profile of a real internal-medicine COPD population; the comorbidity burden of the cohort is quantified by the Charlson comorbidity index in Table 1. All participants were under follow-up at the study centre before enrolment, so baseline records and prior prescriptions were available in the same electronic system. The study was approved by the Scientific Research Ethics Committee of Adana City Training and Research Hospital (decision no. 1138, meeting no. 22, dated 19 February 2026) and conducted in accordance with the Declaration of Helsinki.

### 2.3. Inhaled Treatment and Its Selection

Inhaled treatment was prescribed by the attending physician as part of routine care and was not allocated by the investigators. Each prescribing decision was made individually by the attending internal-medicine specialist for that patient, taking account of GOLD group, symptom burden, exacerbation history, comorbidity, prior treatment and reimbursement status; no treatment board, no study algorithm and no written local protocol governed the choice, and the investigators did not intervene in it. Consequently, participants receiving different regimens differ systematically in baseline severity, and this is quantified in Table 1 and Table 2 and addressed in Section 4. Regimens were recorded at baseline and categorised as follows: SABA/SAMA, a short-acting beta_2_-agonist and/or short-acting muscarinic antagonist with no long-acting maintenance component; LAMA, a long-acting muscarinic antagonist alone; LABA, a long-acting beta_2_-agonist alone; LABA+ICS, a fixed or free combination of a long-acting beta_2_-agonist and an inhaled corticosteroid; LABA+LAMA, dual long-acting bronchodilation; and LABA+LAMA+ICS, triple therapy. The categories are mutually exclusive and reflect the maintenance regimen in place at baseline; as-needed short-acting reliever use was permitted in every category and was not itself a category-defining feature except in the SABA/SAMA group. Because the source records did not distinguish newly initiated from continuing therapy, regimens are described as the baseline maintenance treatment rather than as initial therapy. Non-pharmacological care, vaccination status and pulmonary rehabilitation were recorded but not modified by the study.

### 2.4. Assessments and Outcomes

Participants were assessed at baseline and reassessed at approximately three months. Baseline assessment recorded demographic data, smoking history, anthropometry, comorbidities and the Charlson comorbidity index, blood eosinophil count and exacerbations in the preceding year. Both visits were conducted by the same clinical team using the same instruments and the same equipment.

#### 2.4.1. Spirometry

Spirometry was performed in the hospital pulmonary function laboratory in accordance with the 2019 American Thoracic Society/European Respiratory Society (ATS/ERS) technical statement [17], using a SpiroSphere spirometer (eResearch Technology GmbH, Estenfeld, Germany) subjected to daily volume calibration with a 3 L syringe and to ambient temperature, pressure and humidity correction. All reported values are post-bronchodilator, measured 15 min after 400 µg salbutamol was delivered by metered-dose inhaler. Predicted values were derived from the Global Lung Function 2012 equations [18]. Baseline and follow-up tests were performed in the same laboratory, at a comparable time of day (morning sessions), and under the same medication-withholding instructions: short-acting bronchodilators were withheld for at least 4–6 h and long-acting agents for 24–36 h before testing. The best of at least three acceptable and repeatable manoeuvres was used.

#### 2.4.2. COPD Assessment Test

The validated Turkish version of the CAT was used [19]. The instrument was self-completed by the participant in the clinic before the consultation and before spirometry, so that the score was not influenced by knowledge of the lung-function result. A trained member of the study team read the items aloud and recorded the participant’s own response when literacy or visual impairment prevented self-completion; the response options were never suggested or interpreted by the interviewer. The same procedure was used at both visits. A reduction of at least two points was prespecified as the MCID [11].

#### 2.4.3. Six-Minute Walk Test

The six-minute walk test followed the ERS/ATS technical standard for field walking tests [20]. Tests were conducted along a flat, straight, 30 m indoor corridor marked at both ends, with standardised phrases of encouragement delivered at one-minute intervals and no pacing by the assessor. A practice walk was performed, and where two walks were completed the greater distance was analysed. Supplemental oxygen was administered during the test only to participants already prescribed long-term oxygen therapy, and the same oxygen condition, flow rate and delivery device were used at both visits. A gain of at least 30 m was prespecified as the clinically important improvement threshold [21,22], with a sensitivity analysis at the more conservative threshold of 54 m [23].

#### 2.4.4. mMRC Dyspnoea Scale

Dyspnoea was graded on the modified Medical Research Council (mMRC) scale [24] by the attending study physician during a structured interview using the standard Turkish wording of the five descriptors. The same small group of investigators performed the assessment at both visits and had been briefed on the descriptors before recruitment began; formal inter-rater reliability was not measured, which is acknowledged in Section 4. Because mMRC is an ordinal scale, change was analysed as the paired within-participant difference in grade using the Wilcoxon signed-rank test, and the median grade at each visit is reported alongside the mean change.

#### 2.4.5. Outcomes

The primary outcomes were the within-participant change from baseline to the follow-up visit in CAT score and in FEV_1_ % predicted. Secondary outcomes were the proportion of participants achieving the CAT MCID; the proportion achieving a 6MWD gain of at least 30 m; change in mMRC grade, 6MWD, BODE and ADO indices; and the occurrence of exacerbations, hospitalisation, pneumonia, adverse events, treatment change and treatment discontinuation during follow-up.

### 2.5. Statistical Analysis

Continuous variables are summarised as mean ± standard deviation (SD) or median (interquartile range, IQR) and categorical variables as n (%). Baseline characteristics were compared across the three recorded GOLD groups using one-way analysis of variance, the Kruskal–Wallis test or the chi-square test as appropriate; these comparisons are descriptive and are reported in Table 1 at the reviewers’ request. For each regimen and each GOLD group we report the paired change from baseline to follow-up with a 95% CI, tested with the paired *t*-test for approximately symmetric continuous outcomes and the Wilcoxon signed-rank test for ordinal outcomes and indices. Proportions reaching a threshold are given with Wilson confidence intervals.

Because the study describes the observed course of treated patients rather than testing a prespecified family of competing hypotheses, we report unadjusted *p* values alongside confidence intervals and interpret the size and consistency of the changes rather than significance thresholds alone. Analyses were performed on complete pairs for each outcome; participants missing an outcome at follow-up were excluded from the analysis of that outcome only, no imputation was undertaken, and the number of paired observations is stated for every estimate. Baseline characteristics of participants lost to follow-up were compared with those of participants who completed, and the result is reported in Section 3.1. Regimens are not compared statistically with one another, for the reasons set out in Section 4. Analyses used Python 3.12 with pandas (version 2.1–2.2), NumPy (version 1.26), SciPy (version 1.11–1.14), statsmodels (version 0.14) and Matplotlib (version 3.8–3.9).

## 3. Results

### 3.1. Participants

**Figure 1:**
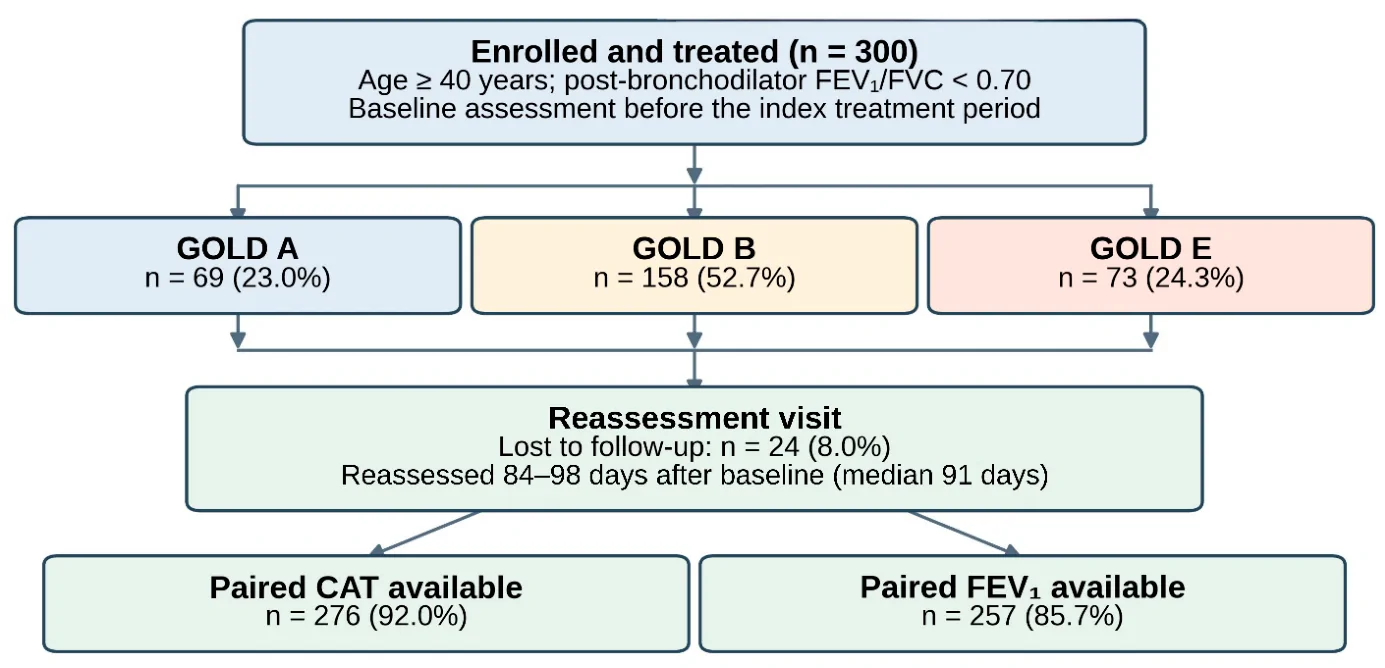
**The participant flow and availability of paired outcome data.** Percentages for the GOLD groups are of the 300 enrolled participants. Paired data refer to participants with both a baseline and a three-month measurement of the outcome concerned. CAT, COPD Assessment Test.

Three hundred participants were enrolled and analysed (Figure 1). Recorded GOLD groups were A, 69 (23.0%); B, 158 (52.7%); and E, 73 (24.3%). Mean age was 62.1 ± 9.0 years and 213 participants (71.0%) were men. Baseline severity increased across the groups, with mean CAT scores of 4.5, 14.9 and 17.5 and mean FEV_1_ of 83.4%, 64.9% and 52.2% predicted in Groups A, B and E respectively (Table 1). Preventive care was incompletely delivered: 109 participants (36.3%) had received influenza vaccination, 64 (21.3%) pneumococcal vaccination and 34 (11.3%) pulmonary rehabilitation.

Paired CAT data were available for 276 participants (92.0%) and paired FEV_1_ for 257 (85.7%); 24 participants (8.0%) were lost to follow-up. All participants who attended the follow-up visit were reassessed between 84 and 98 days after baseline (mean 91.3 ± 4.2 days, median 91); none was reassessed earlier than 84 days. The interval did not differ across GOLD groups (91.5, 91.1 and 91.6 days in Groups A, B and E; *p* = 0.684) or across regimens (range 90.1 to 93.2 days; *p* = 0.120), and it was not correlated with the change in CAT score (r = −0.012). The wider range of 13 to 98 days reported in the submitted version referred to total observed follow-up time including the 24 participants who did not return, whose last recorded contact was a mean of 45.6 days after baseline; it did not describe the reassessment interval of any analysed participant. The wording has been corrected here and in the Conclusions. The most frequently prescribed regimen was LABA+LAMA (n = 116, 38.7%), followed by LAMA (n = 49), LABA+ICS (n = 41), LABA (n = 39), LABA+LAMA+ICS (n = 35) and SABA/SAMA (n = 20).

Participants lost to follow-up had more severe disease at baseline than those who completed: mean FEV_1_ 58.8% versus 66.7% predicted (*p* = 0.040) and mean mMRC grade 2.3 versus 1.5 (*p* = 0.001). Age, CAT score, Charlson index and blood eosinophil count did not differ significantly. Losses were concentrated in Groups B (n = 12) and E (n = 9). The direction of this attrition means that the improvements reported below are likely to be somewhat optimistic, and this is addressed in Section 4.

### 3.2. Change in Symptoms and Lung Function

Across the whole cohort, mean CAT score fell from 13.0 ± 7.1 to 9.1 ± 5.5, a mean reduction of 3.93 points (95% CI −4.43 to −3.44; *p* < 0.001), and one hundred and ninety-five of 276 participants (70.7%, 95% CI 65.0–75.7) achieved the CAT MCID. Mean FEV_1_ % predicted rose by 2.20 percentage points (95% CI 1.78 to 2.63; *p* < 0.001).

**Figure 2:**
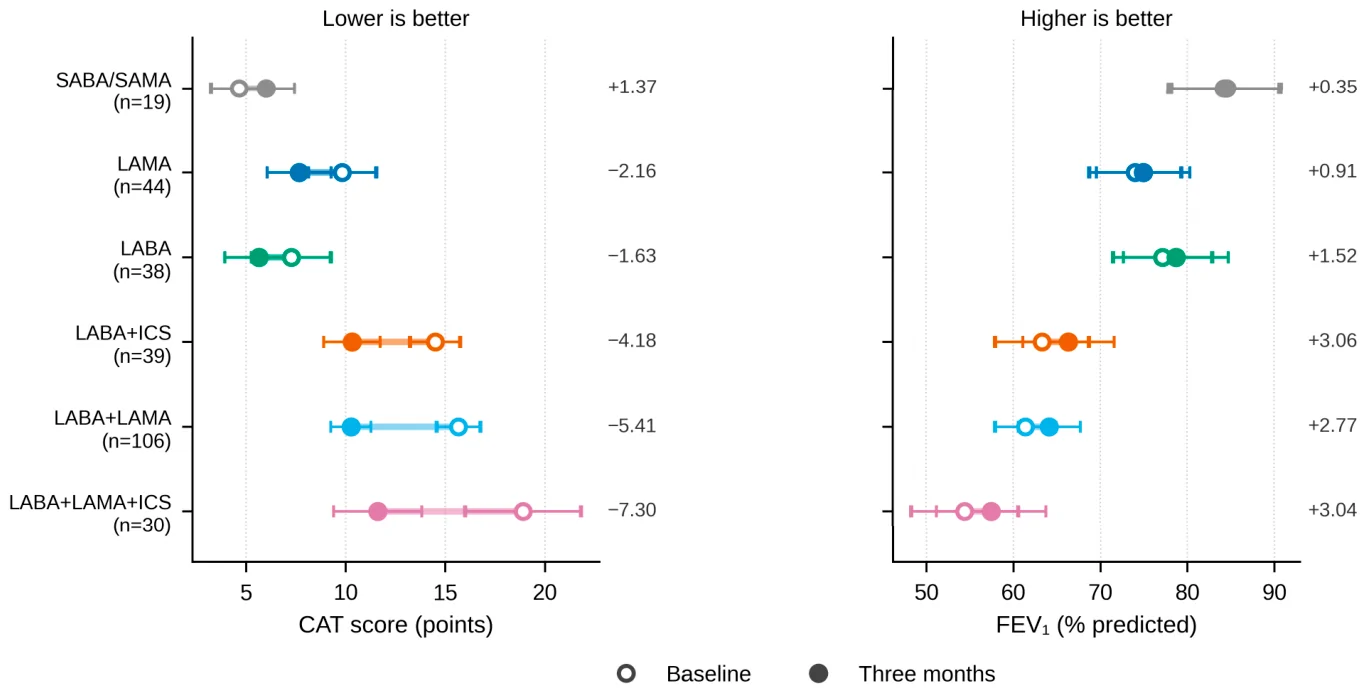
**Mean CAT score and FEV_1_ % predicted at baseline and at three months, by baseline regimen.** The open circles are baseline means and the filled circles are three-month means, joined by a line; the horizontal bars are 95% confidence intervals of the mean. The number beside each row is the mean paired change. n is the number of participants with paired measurements for the outcome concerned. The regimens differed in baseline severity and are not compared with one another; the figure shows the observed course within each treatment group. ICS, inhaled corticosteroid; LABA, long-acting β_2_-agonist; LAMA, long-acting muscarinic antagonist; SABA, short-acting β_2_-agonist; SAMA, short-acting muscarinic antagonist.

**Figure 3:**
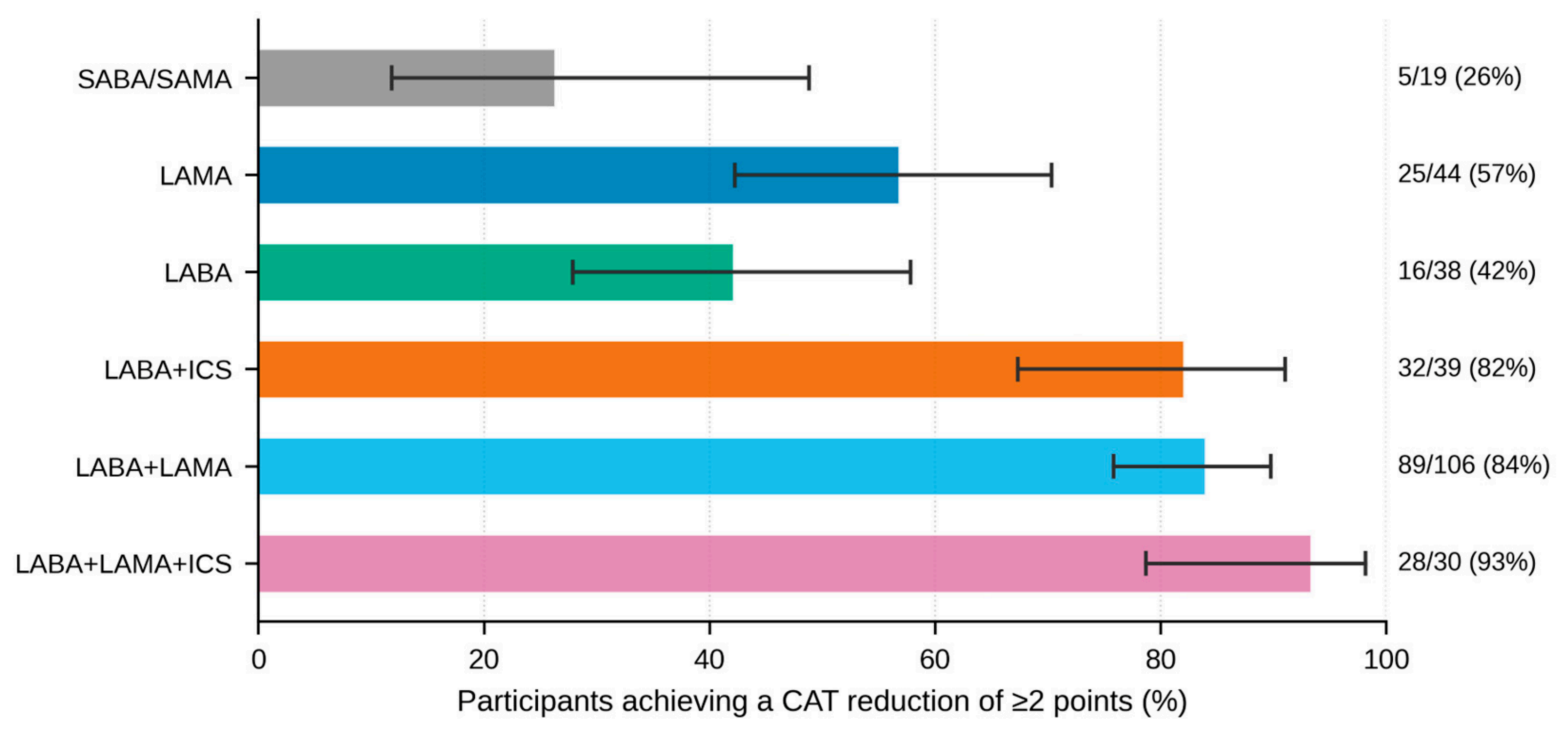
**The proportion of participants achieving the CAT minimal clinically important difference at three months, by baseline regimen.** The minimal clinically important difference is a reduction of at least 2 points in CAT score. The error bars are Wilson 95% confidence intervals. The denominators are participants with paired CAT measurements. As in Figure 2, the groups differed in baseline symptom burden and are not compared statistically.

Improvement was observed with every long-acting regimen and with none of the short-acting treatment (Table 2; Figure 2 and Figure 3). Mean CAT reduction was 2.16 points with LAMA, 1.63 with LABA, 4.18 with LABA+ICS, 5.41 with LABA+LAMA and 7.30 with LABA+LAMA+ICS, all with *p* < 0.001. Participants receiving SABA/SAMA alone showed no improvement, their mean CAT score rising by 1.37 points (95% CI −0.59 to 3.33; *p* = 0.160). Mean FEV_1_ gains followed a similar ordering, from 0.35 percentage points with SABA/SAMA to 3.06 with LABA+ICS, 2.77 with LABA+LAMA and 3.04 with triple therapy.

The proportion of participants reaching the CAT MCID ranged from 26.3% with SABA/SAMA to 93.3% with triple therapy, and exceeded 80% for all three combination regimens: 82.1% (95% CI 67.3–91.0) with LABA+ICS, 84.0% (75.8–89.7) with LABA+LAMA and 93.3% (78.7–98.2) with LABA+LAMA+ICS (Figure 3).

Using the prespecified threshold of 30 m, 98 of 244 participants with paired walk tests (40.2%, 95% CI 34.2–46.4) achieved a clinically important gain in 6MWD. The proportions by regimen were 41.2% with SABA/SAMA, 35.7% with LAMA, 40.0% with LABA, 31.2% with LABA+ICS, 42.6% with LABA+LAMA and 50.0% with triple therapy (Table 3). At the more conservative 54 m threshold the overall proportion fell to 17.6% (95% CI 13.4–22.9). The contrast between the CAT and 6MWD response rates is notable: symptomatic benefit was far more commonly achieved than a clinically important gain in exercise capacity, a divergence discussed in Section 4.

A further point requires emphasis before the regimen-specific figures are read. The regimens differed substantially in baseline severity (Table 1 and Table 2), and those with higher baseline CAT scores had correspondingly more scope for improvement; across the cohort the baseline score was inversely correlated with the subsequent change (r = −0.63). Because treatment was chosen individually according to the very clinical features that also predict the outcome, the ordering of improvements across regimens is confounded by indication and reflects baseline case mix at least as much as any pharmacological contribution. No adjusted or matched comparison between regimens was attempted, and none of the regimen-specific figures should be read as evidence that one regimen outperformed another.

### 3.3. Change Within GOLD Groups

Improvement was concentrated in the symptomatic and exacerbation-prone groups (Table 2). Mean CAT fell by 4.38 points (95% CI −4.94 to −3.81) in Group B and by 6.55 points (95% CI −7.55 to −5.54) in Group E, both *p* < 0.001, whereas Group A showed no significant change (−0.42 points, 95% CI −1.22 to 0.37; *p* = 0.293), consistent with the low baseline symptom burden in that group. FEV_1_ improved in all three groups, by 1.07 percentage points in Group A (*p* = 0.018), 2.64 in Group B and 2.43 in Group E (both *p* < 0.001).

### 3.4. Course of Participants Receiving LABA+ICS

Because current guidance has moved away from LABA+ICS in COPD without asthmatic features, the course of the 41 participants receiving this regimen merits separate description. Their baseline burden was substantial (mean CAT 14.5 ± 4.0; mean FEV_1_ 63.3% predicted). At three months, mean CAT had fallen to 10.3 ± 4.5, a reduction of 4.18 points (95% CI −5.24 to −3.12; *p* < 0.001) that is more than twice the MCID, and 32 of 39 participants (82.1%) met the MCID threshold. Mean FEV_1_ rose by 3.06 percentage points (95% CI 2.16 to 3.95; *p* < 0.001), the largest spirometric gain of any regimen in this cohort. Changes in mMRC grade (0.10, 95% CI −0.11 to 0.31) and 6MWD (5.62 m, 95% CI −10.69 to 21.94) were small and not statistically significant, and only 31.2% achieved the 30 m walk threshold—the lowest proportion of any regimen. One case of pneumonia (2.4%) and two hospitalisations (4.9%) occurred in this group. These are within-group observations in 41 patients selected for this regimen by their physicians; they do not establish that LABA+ICS is equivalent or superior to any alternative.

### 3.5. Secondary Outcomes and Events

Changes in mMRC grade, 6MWD, BODE and ADO indices were smaller and less consistent than those in CAT and FEV_1_ (Table 3). 6MWD increased by 20.0 m (95% CI 12.4 to 27.7; *p* < 0.001) in the LABA+LAMA group and by similar point estimates in most other groups, though with wider intervals. BODE and ADO indices improved modestly in the LABA+LAMA group and changed little elsewhere.

During follow-up, sixty-three exacerbations occurred in 55 participants, together with eleven hospitalisations, four cases of pneumonia, thirty-six adverse events, thirty-seven treatment changes and sixteen treatment discontinuations (Table 4). Events were most frequent in the groups with the highest baseline risk.

## 4. Discussion

### 4.1. Main Findings

In this prospective cohort of 300 patients managed in an internal-medicine clinic, approximately three months of individually tailored inhaled therapy was accompanied by a mean CAT reduction of almost four points and a mean FEV_1_ gain of 2.2 percentage points, with seven of ten participants reaching the CAT minimal clinically important difference. Improvement was evident with every long-acting regimen and in the symptomatic GOLD B and E groups, and was absent among participants treated with short-acting bronchodilators alone. Gains in exercise capacity were more modest: fewer than half of participants achieved the 30 m threshold for a clinically important improvement in 6MWD, and fewer than one in five reached 54 m. Symptom relief and measurable improvement in walking distance are therefore not interchangeable endpoints in this setting.

The pattern is clinically coherent. Patients with little symptom burden at baseline (Group A, mean CAT 4.5) had limited scope to improve and did not do so, whereas those entering the study with substantial burden improved substantially. Taken together with the absence of change under SABA/SAMA, this is consistent with the routine practice of establishing long-acting maintenance therapy in symptomatic patients rather than relying on rescue treatment alone, although the uncontrolled design means the observation cannot on its own establish that relationship.

### 4.2. Course of Patients Receiving LABA+ICS

Participants receiving LABA+ICS improved by more than twice the CAT minimal clinically important difference, more than four in five reached the MCID threshold, and their mean FEV_1_ gain of 3.06 percentage points was the largest recorded in this cohort. These figures are of similar magnitude to those observed on LABA+LAMA and on triple therapy, and indicate that patients maintained on a LABA–ICS combination in routine care experienced symptomatic and spirometric improvement of a magnitude that exceeded the established MCID.

This observation deserves careful rather than enthusiastic interpretation. Guidance has moved away from LABA+ICS for COPD without asthmatic features, on the basis of randomised evidence that dual bronchodilation performs better in exacerbation-prone patients [5] and that triple therapy is the preferred route when an ICS is indicated [2,8]. Nothing in an uncontrolled cohort can overturn that evidence, and our design does not permit us to say that LABA+ICS performed as well as, better than, or worse than any comparator; the participants who received it differed from those who received other regimens in ways that we did not attempt to adjust away. What these data do show is that the substantial group of patients still maintained on LABA+ICS in everyday practice—41 participants, 13.7% of this cohort—were not stagnating: their symptoms and spirometry moved in the expected direction and by an amount exceeding the MCID over three months, even though their gain in walking distance was the smallest of any regimen. For clinicians managing such patients, this is a reassuring rather than a prescriptive finding, and it does not displace the guideline recommendation to review ICS indication, asthmatic features and eosinophil count when treatment is reassessed. The pneumonia risk associated with ICS exposure [25] also remains a consideration that a three-month cohort with four cases of pneumonia cannot address.

### 4.3. Comparison with Other Real-World Data

The magnitude of symptomatic improvement we observed is broadly consistent with recent real-world studies of inhaled therapy in COPD, which have generally reported improvements in health status exceeding the MCID after treatment is established or escalated [14,15]. Those studies share our principal interpretive limitation: improvement measured before and after a treatment decision reflects the natural history of a selected group as well as any pharmacological effect.

### 4.4. Inhaler Device, Technique and Inspiratory Flow

Device selection and inhalation technique are central to the effectiveness of any inhaled regimen, and critical errors are common and associated with worse symptom control and more frequent exacerbations [26]. The present study did not record the device type dispensed within each regimen category, the reasons for device choice, peak inspiratory flow, or the results of any technique assessment, and it did not capture whether device switching occurred during follow-up. Inhaler education and demonstration form part of routine practice at the study centre, but this was not delivered according to a study protocol, was not documented in a structured way, did not employ a formal teach-back procedure, and was not reassessed at follow-up. These data are therefore unavailable and cannot be reconstructed retrospectively. This is a substantive limitation: some of the improvement observed here may reflect correction of inhaler technique at the baseline consultation rather than the pharmacological properties of the regimen prescribed, and differences between the regimens may partly reflect differences in the devices through which they were delivered. Prospective recording of device type, technique score and peak inspiratory flow is a priority for future work in this setting.

### 4.5. Assessment Interval, Treatment Status and the CAT Recall Period

All analysed participants were reassessed between 84 and 98 days after baseline, so the interval was narrow and did not differ significantly by GOLD group or regimen, and it was uncorrelated with the change in CAT score. The four-week recall period of the CAT therefore fell entirely within the treatment interval for every participant contributing paired data, and no CAT score was obtained before the recall window had elapsed. The broader 13 to 98 day range quoted in the submitted version described total observed follow-up time including participants who never returned; that wording was imprecise and has been corrected.

A more consequential limitation concerns baseline treatment status. The source records did not distinguish participants who were treatment-naive at enrolment from those continuing, switching, escalating or de-escalating an existing regimen, and this variable cannot be recovered from the data collected. The distinction matters: treatment-naive patients have greater scope for symptomatic and spirometric improvement than patients already established on maintenance therapy, and prescribing decisions are themselves shaped by treatment history. Since regimen groups almost certainly differ in the proportion of treatment-naive patients they contain, some of the variation in improvement across regimens may reflect differing treatment histories rather than differing pharmacology. This is one of the principal reasons why we have not compared regimens statistically. Similarly, changes in smoking status during follow-up, uptake of pulmonary rehabilitation, adherence, and concurrent adjustments to non-inhaled treatment were not captured, and any of these could have contributed to the observed changes.

### 4.6. Limitations

Several limitations must be considered together, and no single one of them can be set above the others. The study had no concurrent control group. A cohort followed under a randomised design, or an untreated or differently treated comparator group, would have permitted causal inference; without one, the observed changes cannot be attributed to the inhaled regimens. Regression to the mean, which was substantial here (r = −0.63 between baseline CAT and subsequent change); the resolution of subclinical worsening around the time of a clinic visit; improved adherence and inhaler technique after review; seasonal variation; and the effect of study participation itself may all contribute. For the same reason we have refrained from comparing the regimens with one another; because prescribing was determined by the same clinical features that predict the outcome, such comparisons in observational data can diverge substantially from randomised findings [27].

The observation period of approximately three months is short. It is sufficient to capture change in symptoms and spirometry but too brief to assess exacerbation prevention, durability of benefit, or mortality, and the sixty-three exacerbations, eleven hospitalisations and four cases of pneumonia recorded are too few to support any inference about comparative safety. Further limitations include the single-centre internal-medicine setting, which may not generalise to pulmonology or primary-care populations; the absence of data on adherence, inhaler technique, device, dose and molecule; the availability of only a single baseline eosinophil measurement; the absence of formal inter-rater reliability testing for the mMRC grading; and the loss to follow-up of 8.0% of participants, which was greater among patients with lower FEV_1_ and higher mMRC grade and could therefore bias the observed improvement upwards. Finally, the analysis is descriptive and involves multiple outcomes; individual *p* values are presented without correction for multiplicity and should be read as supporting the description rather than as formal hypothesis tests.

### 4.7. Future Directions

The limitations above define a clear agenda. A prospective study capable of answering the comparative question would need a concurrent control group, ideally through randomisation of treatment strategy or, where randomisation is impractical, through a target-trial emulation with propensity-score matching and a defined new-user design that separates treatment-naive patients from prevalent users. Device type, formal inhalation-technique scoring with teach-back, peak inspiratory flow and objective adherence measurement should be collected at every visit. Follow-up should extend to at least twelve months to capture exacerbations and durability, with serial rather than single eosinophil measurements, and recruitment should span several centres and both internal-medicine and pulmonology settings. A multicentre registry built on these principles would also permit the analysis of care gaps that this cohort only describes, such as the low uptake of vaccination and pulmonary rehabilitation.

## 5. Conclusions

Over a reassessment interval of 84 to 98 days, patients with COPD receiving individually tailored inhaled therapy in an internal-medicine clinic showed improvement in symptoms and lung function that exceeded the established minimal clinically important difference for the CAT, with around 70% of participants reaching that threshold; gains in exercise capacity were more modest, with 40% reaching the 30 m threshold for the six-minute walk distance. Improvement was observed with every long-acting regimen, including LABA+ICS, and was concentrated in the symptomatic GOLD B and E groups, whereas participants receiving short-acting bronchodilators alone did not improve. These are within-patient changes observed in a single-centre cohort without randomisation, without a concurrent control group and without adjustment for the substantial baseline differences between regimen groups. They therefore characterise the course of treated patients and must not be read as evidence that any regimen is superior to another; that question requires randomised or appropriately designed comparative studies.

## Figures and Tables

**Table 1:** Baseline characteristics by recorded GOLD group.

Characteristic	All Participants (n = 300)	GOLD A (n = 69)	GOLD B (n = 158)	GOLD E (n = 73)	*p*
Age, years	62.1 ± 9.0	55.9 ± 8.3	62.7 ± 8.5	66.8 ± 7.4	<0.001
Male sex, n (%)	213 (71.0)	50 (72.5)	111 (70.3)	52 (71.2)	0.943
Body mass index, kg/m^2^	28.3 ± 4.9	30.3 ± 4.4	28.3 ± 4.6	26.6 ± 5.3	<0.001
Current smoker, n (%)	101 (33.7)	27 (39.1)	49 (31.0)	25 (34.2)	0.489
Smoking exposure, pack-years	36.6 (18.5–46.4)	26.9 (14.9–39.6)	36.2 (19.2–46.5)	43.0 (24.6–53.4)	0.001
Charlson comorbidity index	1.0 (1.0–2.0)	1.0 (1.0–2.0)	1.0 (1.0–2.0)	2.0 (1.0–3.0)	<0.001
FEV_1_, % predicted	66.0 ± 18.6	83.4 ± 11.6	64.9 ± 16.1	52.2 ± 16.1	<0.001
FEV_1_/FVC, %	56.8 ± 5.9	60.0 ± 5.0	56.6 ± 5.6	54.0 ± 6.0	<0.001
CAT score	13.2 ± 7.2	4.5 ± 2.9	14.9 ± 5.2	17.5 ± 7.2	<0.001
mMRC dyspnoea grade	2.0 (1.0–2.0)	0.0 (0.0–1.0)	2.0 (1.0–2.0)	3.0 (2.0–3.0)	<0.001
6MWD, m	447.0 ± 98.0	523.3 ± 76.4	440.0 ± 85.9	382.5 ± 91.0	<0.001
BODE index	1.0 (0.0–3.0)	0.0 (0.0–0.0)	1.0 (1.0–3.0)	3.0 (2.0–5.0)	<0.001
ADO index	4.0 (2.0–6.0)	2.0 (1.0–2.0)	4.0 (2.0–6.0)	6.0 (5.0–8.0)	<0.001
Blood eosinophils, cells/µL	172.5 (100.0–293.0)	184.0 (100.0–293.0)	170.5 (97.2–294.5)	158.0 (115.0–282.0)	0.787
Blood eosinophils ≥300 cells/µL, n (%)	72 (24.0)	17 (24.6)	38 (24.1)	17 (23.3)	0.982
Moderate exacerbations, previous year	0.0 (0.0–1.0)	0.0 (0.0–0.0)	0.0 (0.0–1.0)	2.0 (1.0–2.0)	<0.001
Influenza vaccination, n (%)	109 (36.3)	12 (17.4)	49 (31.0)	48 (65.8)	<0.001
Pneumococcal vaccination, n (%)	64 (21.3)	22 (31.9)	21 (13.3)	21 (28.8)	0.001
Pulmonary rehabilitation, n (%)	34 (11.3)	7 (10.1)	21 (13.3)	6 (8.2)	0.496

Values are mean ± SD, median (IQR) or n (%). *p*-values compare the three recorded GOLD groups by one-way analysis of variance (means), the Kruskal–Wallis test (medians) or the chi-square test (proportions). These comparisons describe the recorded groups; they are not tests of any analytic contrast, since no between-regimen comparison was performed. 6MWD and BODE index were missing for 23 participants and pack-years for 5. ADO, age–dyspnoea–obstruction; BODE, body mass index–airflow obstruction–dyspnoea–exercise capacity; CAT, COPD Assessment Test; FEV_1_, forced expiratory volume in one second; FVC, forced vital capacity; IQR, interquartile range; mMRC, modified Medical Research Council; 6MWD, six-minute walk distance; SD, standard deviation.

**Table 2:** Change in CAT score and FEV_1_ % predicted from baseline to three months.

		CAT Score						FEV_1_ % Predicted				
	Total n	Paired n	Baseline	3 Months	Mean Change (95% CI)	*p*	MCID Reached n/N (%; 95% CI)	Paired n	Baseline	3 Months	Mean Change (95% CI)	*p*
**Panel A. By baseline regimen**												
SABA/SAMA	20	19	4.6 ± 3.1	6.0 ± 3.2	1.37 (−0.59 to 3.33)	0.160	5/19 (26.3; 11.8–48.8)	18	84.2 ± 13.8	84.5 ± 13.6	0.35 (−1.67 to 2.37)	0.720
LAMA	49	44	9.8 ± 5.8	7.7 ± 5.4	−2.16 (−3.08 to −1.24)	<0.001	25/44 (56.8; 42.2–70.3)	40	74.0 ± 17.1	74.9 ± 17.3	0.91 (−0.29 to 2.11)	0.134
LABA	39	38	7.3 ± 6.2	5.6 ± 5.4	−1.63 (−2.55 to −0.72)	<0.001	16/38 (42.1; 27.9–57.8)	36	77.2 ± 17.5	78.7 ± 18.4	1.52 (0.24 to 2.81)	0.021
LABA+ICS	41	39	14.5 ± 4.0	10.3 ± 4.5	−4.18 (−5.24 to −3.12)	<0.001	32/39 (82.1; 67.3–91.0)	35	63.3 ± 16.3	66.4 ± 15.9	3.06 (2.16 to 3.95)	<0.001
LABA+LAMA	116	106	15.7 ± 5.7	10.3 ± 5.3	−5.41 (−6.14 to −4.67)	<0.001	89/106 (84.0; 75.8–89.7)	100	61.4 ± 17.8	64.1 ± 18.2	2.77 (2.09 to 3.45)	<0.001
LABA+LAMA+ICS	35	30	18.9 ± 8.1	11.6 ± 6.2	−7.30 (−8.66 to −5.94)	<0.001	28/30 (93.3; 78.7–98.2)	28	54.4 ± 16.6	57.5 ± 17.0	3.04 (2.07 to 4.00)	<0.001
**All participants**	**300**	**276**	**13.0 ± 7.1**	**9.1 ± 5.5**	**−3.93 (−4.43 to −3.44)**	**<0.001**	**195/276 (70.7; 65.0–75.7)**	**257**	**66.7 ± 19.0**	**68.9 ± 18.9**	**2.20 (1.78 to 2.63)**	**<0.001**
**Panel B. By recorded GOLD group**												
GOLD A	69	66	4.4 ± 2.9	4.0 ± 3.3	−0.42 (−1.22 to 0.37)	0.293	23/66 (34.8; 24.5–46.9)	64	83.4 ± 11.9	84.5 ± 11.8	1.07 (0.19 to 1.96)	0.018
GOLD B	158	146	14.9 ± 4.9	10.5 ± 5.1	−4.38 (−4.94 to −3.81)	<0.001	114/146 (78.1; 70.7–84.0)	134	65.0 ± 16.6	67.7 ± 17.2	2.64 (2.04 to 3.25)	<0.001
GOLD E	73	64	17.6 ± 7.1	11.1 ± 5.3	−6.55 (−7.55 to −5.54)	<0.001	58/64 (90.6; 81.0–95.6)	59	52.2 ± 16.5	54.6 ± 16.3	2.43 (1.64 to 3.23)	<0.001

Values are mean ± SD in participants with paired measurements. Mean change is the paired within-participant difference (three months minus baseline) with a t-based 95% confidence interval; *p* values are from paired *t*-tests and are not corrected for multiplicity. Negative CAT and positive FEV_1_ changes indicate improvement. MCID, minimal clinically important difference, defined as a reduction of at least 2 points in CAT score, with Wilson 95% confidence intervals. The groups differed in baseline severity and are not compared with one another; these are within-group changes over time in an uncontrolled cohort. CAT, COPD Assessment Test; CI, confidence interval; FEV_1_, forced expiratory volume in 1 s; GOLD, Global Initiative for Chronic Obstructive Lung Disease; ICS, inhaled corticosteroid; LABA, long-acting β_2_-agonist; LAMA, long-acting muscarinic antagonist; SABA, short-acting β_2_-agonist; SAMA, short-acting muscarinic antagonist; SD, standard deviation.

**Table 3:** Change in secondary outcomes from baseline to three months, by baseline regimen.

Outcome	Baseline Regimen	Paired n	Baseline	3 Months	Mean Change (95% CI)	*p*
mMRC dyspnoea grade	SABA/SAMA	19	0.0 (0.0–1.0)	1.0 (0.0–1.0)	0.26 (0.05 to 0.48)	0.025
mMRC dyspnoea grade	LAMA	44	1.0 (0.0–2.0)	1.0 (0.0–2.0)	0.05 (−0.14 to 0.23)	0.617
mMRC dyspnoea grade	LABA	38	1.0 (0.0–1.0)	0.0 (0.0–1.0)	−0.05 (−0.25 to 0.15)	0.593
mMRC dyspnoea grade	LABA + ICS	39	2.0 (1.0–2.5)	2.0 (1.0–3.0)	0.10 (−0.11 to 0.31)	0.317
mMRC dyspnoea grade	LABA + LAMA	106	2.0 (1.0–3.0)	2.0 (1.0–3.0)	−0.13 (−0.27 to 0.00)	0.057
mMRC dyspnoea grade	LABA + LAMA + ICS	30	2.0 (1.0–4.0)	2.0 (1.0–3.0)	−0.13 (−0.39 to 0.12)	0.285
**mMRC dyspnoea grade**	**All participants**	**276**	**1.0 (0.0–2.0)**	**1.0 (0.0–2.0)**	**−0.03 (−0.11 to 0.05)**	**0.409**
Six-minute walk distance, m	SABA/SAMA	17	523.8 ± 62.4	544.2 ± 71.0	20.47 (0.82 to 40.12)	0.042
Six-minute walk distance, m	LAMA	42	477.2 ± 94.1	489.2 ± 91.6	12.00 (−1.95 to 25.95)	0.090
Six-minute walk distance, m	LABA	35	503.6 ± 102.6	522.7 ± 121.2	19.17 (2.44 to 35.90)	0.026
Six-minute walk distance, m	LABA + ICS	32	444.6 ± 75.0	450.2 ± 93.6	5.62 (−10.69 to 21.94)	0.487
Six-minute walk distance, m	LABA + LAMA	94	418.6 ± 90.9	438.6 ± 95.5	20.03 (12.40 to 27.66)	<0.001
Six-minute walk distance, m	LABA + LAMA + ICS	24	383.2 ± 84.6	400.0 ± 80.0	16.75 (−1.44 to 34.94)	0.069
**Six-minute walk distance, m**	**All participants**	**244**	**448.1 ± 97.6**	**464.5 ± 103.7**	**16.34 (11.05 to 21.64)**	**<0.001**
BODE index	SABA/SAMA	17	0.0 (0.0–0.0)	0.0 (0.0–0.0)	0.12 (−0.05 to 0.29)	0.157
BODE index	LAMA	39	1.0 (0.0–2.0)	0.0 (0.0–2.0)	0.03 (−0.19 to 0.24)	0.813
BODE index	LABA	33	0.0 (0.0–0.0)	0.0 (0.0–0.0)	−0.06 (−0.28 to 0.16)	0.564
BODE index	LABA + ICS	28	1.5 (1.0–2.2)	2.0 (0.8–3.0)	0.00 (−0.30 to 0.30)	1.000
BODE index	LABA + LAMA	83	2.0 (1.0–4.0)	2.0 (0.5–3.0)	−0.30 (−0.48 to −0.12)	0.001
BODE index	LABA + LAMA + ICS	21	3.0 (1.0–5.0)	2.0 (1.0–5.0)	−0.05 (−0.54 to 0.44)	0.748
**BODE index**	**All participants**	**221**	**1.0 (0.0–3.0)**	**1.0 (0.0–3.0)**	**−0.11 (−0.21 to −0.01)**	**0.028**
ADO index	SABA/SAMA	19	2.0 (1.0–2.5)	2.0 (1.0–3.0)	0.26 (0.05 to 0.48)	0.025
ADO index	LAMA	44	3.0 (1.0–4.2)	3.0 (1.0–5.0)	0.05 (−0.19 to 0.28)	0.697
ADO index	LABA	38	2.0 (1.0–3.0)	2.0 (1.0–3.0)	−0.11 (−0.33 to 0.12)	0.346
ADO index	LABA + ICS	39	4.0 (2.5–6.5)	4.0 (2.0–6.0)	−0.08 (−0.33 to 0.17)	0.532
ADO index	LABA + LAMA	106	5.0 (3.0–6.0)	4.5 (3.0–6.0)	−0.28 (−0.44 to −0.12)	<0.001
ADO index	LABA + LAMA + ICS	30	6.0 (4.0–7.8)	6.0 (3.0–7.0)	−0.27 (−0.59 to 0.06)	0.096
**ADO index**	**All participants**	**276**	**4.0 (2.0–6.0)**	**4.0 (2.0–6.0)**	**−0.14 (−0.23 to −0.04)**	**0.004**
6MWD gain ≥30 m, n/N (%; 95% CI)	SABA/SAMA	17	–	–	7/17 (41.2; 21.6–64.0)	–
6MWD gain ≥30 m, n/N (%; 95% CI)	LAMA	42	–	–	15/42 (35.7; 23.0–50.8)	–
6MWD gain ≥30 m, n/N (%; 95% CI)	LABA	35	–	–	14/35 (40.0; 25.6–56.4)	–
6MWD gain ≥30 m, n/N (%; 95% CI)	LABA + ICS	32	–	–	10/32 (31.2; 18.0–48.6)	–
6MWD gain ≥30 m, n/N (%; 95% CI)	LABA + LAMA	94	–	–	40/94 (42.6; 33.0–52.6)	–
6MWD gain ≥30 m, n/N (%; 95% CI)	LABA + LAMA + ICS	24	–	–	12/24 (50.0; 31.4–68.6)	–
**6MWD gain ≥30 m, n/N (%; 95% CI)**	**All participants**	**244**	**–**	**–**	**98/244 (40.2; 34.2–46.4)**	**–**
**6MWD gain ≥54 m, n/N (%; 95% CI)**	**All participants**	**244**	**–**	**–**	**43/244 (17.6; 13.4–22.9)**	**–**

The baseline and three-month values are the median (interquartile range) except for six-minute walk distance, which is mean ± SD. The mean change is the paired arithmetic difference with a t-based 95% confidence interval; *p* values are from Wilcoxon signed-rank tests, uncorrected for multiplicity. These are within-group changes over time and do not compare regimens. Clinically important gains in six-minute walk distance are shown as n/N (%; Wilson 95% CI) at the prespecified threshold of 30 m and, as a sensitivity analysis, at 54 m (whole cohort). ADO, age–dyspnoea–obstruction index; BODE, body-mass index, airflow obstruction, dyspnoea and exercise capacity index; CI, confidence interval; mMRC, modified Medical Research Council; ICS, inhaled corticosteroid; LABA, long-acting β_2_-agonist; LAMA, long-acting muscarinic antagonist; SABA, short-acting β_2_-agonist; SAMA, short-acting muscarinic antagonist; SD, standard deviation; 6MWD, six-minute walk distance.

**Table 4:** Clinical events during follow-up, by baseline regimen.

Baseline Regimen	n	Exacerbations, Total	Hospitalisation n (%)	Pneumonia n (%)	Any Adverse Event n (%)	Treatment Change n (%)	Discontinuation n (%)
SABA/SAMA	20	4	1 (5.0)	1 (5.0)	3 (15.0)	3 (15.0)	0 (0.0)
LAMA	49	1	0 (0.0)	1 (2.0)	8 (16.3)	2 (4.1)	3 (6.1)
LABA	39	6	2 (5.1)	1 (2.6)	5 (12.8)	5 (12.8)	2 (5.1)
LABA + ICS	41	12	2 (4.9)	1 (2.4)	9 (22.0)	5 (12.2)	4 (9.8)
LABA + LAMA	116	26	3 (2.6)	0 (0.0)	9 (7.8)	16 (13.8)	6 (5.2)
LABA + LAMA + ICS	35	14	3 (8.6)	0 (0.0)	2 (5.7)	6 (17.1)	1 (2.9)
**All participants**	**300**	**63**	**11 (3.7)**	**4 (1.3)**	**36 (12.0)**	**37 (12.3)**	**16 (5.3)**

Exacerbations are the total number of events recorded during follow-up; all other columns are the number and percentage of participants with at least one event. Events were more frequent in groups with greater baseline severity, and these descriptive counts are not adjusted for that difference. Events were recorded up to the reassessment visit (84–98 days after baseline) or, for the 24 participants lost to follow-up, up to the last recorded contact. ICS, inhaled corticosteroid; LABA, long-acting β_2_-agonist; LAMA, long-acting muscarinic antagonist; SABA, short-acting β_2_-agonist; SAMA, short-acting muscarinic antagonist.

## Data Availability

The de-identified dataset is available from the corresponding author on reasonable request, subject to institutional approval.
